# CgEnd3 Regulates Endocytosis, Appressorium Formation, and Virulence in the Poplar Anthracnose Fungus *Colletotrichum gloeosporioides*

**DOI:** 10.3390/ijms22084029

**Published:** 2021-04-14

**Authors:** Xiaolian Wang, Dongxiao Lu, Chengming Tian

**Affiliations:** The Key Laboratory for Silviculture and Conservation of Ministry of Education, College of Forestry, Beijing Forestry University, Beijing 100083, China; XiaolianWang94@163.com (X.W.); ludongxiaou@163.com (D.L.)

**Keywords:** anthracnose, *Colletotrichum gloeosporioides*, appressorium, endocytosis, pathogenicity, calcium signaling

## Abstract

The hemibiotrophic ascomycete fungus *Colletotrichum gloeosporioides* is the causal agent of anthracnose on numerous plants, and it causes considerable economic losses worldwide. Endocytosis is an essential cellular process in eukaryotic cells, but its roles in *C. gloeosporioides* remain unknown. In our study, we identified an endocytosis-related protein, CgEnd3, and knocked it out via polyethylene glycol (PEG)-mediated protoplast transformation. The lack of *CgEnd3* resulted in severe defects in endocytosis. *C. gloeosporioides* infects its host through a specialized structure called appressorium, and Δ*CgEnd3* showed deficient appressorium formation, melanization, turgor pressure accumulation, penetration ability of appressorium, cellophane membrane penetration, and pathogenicity. *CgEnd3* also affected oxidant adaptation and the expression of core effectors during the early stage of infection. CgEnd3 contains one EF hand domain and four calcium ion-binding sites, and it is involved in calcium signaling. A lack of *CgEnd3* changed the responses to cell-wall integrity agents and fungicide fludioxonil. However, *CgEnd3* regulated appressorium formation and endocytosis in a calcium signaling-independent manner. Taken together, these results demonstrate that *CgEnd3* plays pleiotropic roles in endocytosis, calcium signaling, cell-wall integrity, appressorium formation, penetration, and pathogenicity in *C. gloeosporioides*, and it suggests that *CgEnd3* or endocytosis-related genes function as promising antifungal targets.

## 1. Introduction

*Colletotrichum* species are known to cause anthracnose on a wide variety of host plants, including cereals, fruit plants, legumes, vegetables, fruit trees, and forest trees [1]. Poplar (*Populus × beijingensis*) is among the most commonly cultivated forestry species in China, and the hemibiotrophic fungus *Colletotrichum gloeosporioides* is the causal agent of poplar anthracnose, which causes considerable economic losses [2,3]. This fungus forms a specialized infection structure, called an appressorium, which correlates strongly with pathogenicity [3,4,5]. The poplar anthracnose infection initiates with the adhesion of the conidium on the leaf surface; then, germ tubes form from the germinated conidium [6]. Various host signals, including epicuticular waxes, cutin monomers, hydrophobicity, hardness, and topography, are recognized at this stage, and appressorium is subsequently formed at the tip of the germ tube [7]. A mature appressorium mechanically and enzymatically ruptures the cuticles of leaves. Then, the penetration peg at the infection site differentiates into the primary hyphae, which grow into the epidermal cells, and secondary hyphae, which subsequently spread through the underlying mesophyll cells and develop necrotic lesions [6].

Appressoria are specialized infection structures in many phytopathogens, which play a key role in the penetration of the host cuticle. There are many studies that focus on the regulation of appressorium development in model pathogenic fungi, including *Magnaporthe oryzae*, *Botrytis cinerea*, rust fungi, *Ustilago maydis*, and *Colletotrichum* species [5]. At the early stage of appressorium development, cell surface sensors recognize the host signals and subsequently activate downstream pathways, such as the Cyclic adenosine monophosphate (cAMP) pathway and mitogen-activated kinase pathway [8,9,10,11]. In addition to the perception of host signals, cell cycle control plays an important role in appressorium development. In *M. oryzae* and *U. maydis*, the intervention of the cell cycle checkpoint during mitosis prevented both appressorium development and autophagy [12,13]. Autophagy in germinated conidia is a prerequisite for the degradation of intracellular contents, which contributes to the accumulation of turgor pressure [14]. During the maturation of appressoria, the inner side of the appressorium cell wall forms a melanin layer, which provides the structural rigidity of appressorium [15]. Mature appressorium mechanically and enzymatically ruptures the cuticle of the host surface, and penetration pegs form subsequently. The turgor pressure provides the essential driving force that enables the fungal peg to mechanically penetrate the host tissue [16], and the melanin deposit in the cell wall of appressorium is essential for the maintenance of high turgor pressure [17,18]. In *Colletotrichum higginsianum*, random insertional mutagenesis identified six mutants that were impaired in appressoria melanization, and five out of six mutants are completely unable to penetrate living host epidermal cells, ethanol-killed leaves, or cellophane membranes [19]. In *Colletotrichum orbiculare*, transcripts of three melanin biosynthesis genes, *PKS1*, *SCD1*, and *THR1*, are increased during appressorium differentiation [20]. The deletion of *PKS1*, *SCD1,* and *THR1* affects the melanization of appressoria, and it results in the penetration defect and decreased virulence [21,22,23]. In *C. gloeosporioides*, the deletion of *CgScd1* results in complete loss of melanization in appressoria, impaired infection ability of nonmelanized appressoria, and the virulence of Δ*CgScd1* is significantly decreased in tomato and lime fruits [24]. These studies indicated that the melanin deposition in appressoria plays a vital role in penetration and pathogenicity. In *C. higginsianum*, *C. orbiculare,* and *M. oryzae*, a variety of effectors are secreted during the infection process to suppress host immunity [25,26,27]. Collectively, the formation of appressorium and subsequent penetration involves a subset of molecular processes [28], and the characterization of appressorium-related genes is vital for the identification of these molecular mechanisms.

In eukaryotic cells, the intracellular calcium ion plays an important role in cellular processes, and it is required for the response to diverse environmental cues [29]. Cytosolic Ca^2+^ binds to and activates the protein calmodulin, which contains the Ca^2+^-binding motif α-helix E loop α-helix F (EF) hands, and subsequently activates calmodulin-dependent enzymes, such as calcineurin, calmodulin-dependent protein kinases, and histone deacetylases [30,31]. Calcium signaling plays crucial roles in stress tolerance, sporulation, cell-wall integrity, and pathogenicity in fungi [32,33,34]. In *M. oryzae*, the calcineurin-responsive transcription factor, *MoCrz1*, regulates calcium ion response, vegetative growth, cell-wall integrity, and pathogenicity [35]. *VdCrz1* regulates cell-wall integrity, microsclerotia formation, and pathogenicity in *Verticillium dahliae* [36]. *BcCrz1* is involved in stress response, cell-wall integrity, sclerotia formation, and pathogenicity in *B. cinerea* [37]. In *U. maydis,* the deletion of endoplasmic calcium ATPase *Eca1* results in severe growth and morphology defects [38]. In *M. oryzae*, the primary functions of almost all known calcium-signaling proteins in the genome are characterized using the high-throughput RNA silencing system, and the results show that 26, 35, and 15 out of the 37 calcium signaling-related genes are involved in vegetative growth, sporulation, and pathogenicity, respectively [39].

In the eukaryotic cell, the optimal growth requires the elaboration of responses to the extracellular environment, and endocytosis is an important process for the maintenance of cellular homeostasis. Endocytosis is an important cellular process in the eukaryotic cell, which internalizes membrane proteins and macromolecules from the extracellular environment [40]. Nevertheless, the molecular studies of endocytosis in fungi has only proceeded in last two decades, with the development of the fluorescent dye N-(3-triethylammoniumpropyl)-4-(p-diethylaminophenylhexatrienyl)-pyridinium 2Br (FM4-64) as an indicator of endocytosis [41]. The systematic mechanism of endocytosis has been well illuminated in the yeast, *Saccharomyces cerevisiae*, such as the role of *END4/SLA2* [42,43]. In any case, Snf1 acts as a nutrient-sensing regulator of endocytosis [44]. In addition, End3p is a key regulator of the organization of cortical actin cytoskeleton and endocytosis, and a lack of *End3p* affects the internalization of a variety of yeast plasma membrane proteins [45,46]. *End3p* is also required for the correct distribution of chitin at the cell surface [47]. Despite the well-developed network of endocytosis in yeast, the mechanisms of endocytosis in *Colletotrichum* species are basically unknown. In this study, we identified *CgEnd3* in *C. gloeosporioides*, which isorthologous to yeast *End3p*. In this study, *CgEnd3* is a key regulator of endocytosis, and the deletion of *CgEnd3* resulted in the significantly decreased ability of appressorium formation and penetration. The pathogenicity of the *CgEnd3* deletion mutant is significantly decreased compared with that of the wild type (WT), which is attributed to the defects in oxidant adaptation and the expression of core effectors. Our study firstly reports on the role of *CgEnd3* in calcium signaling. In addition, *CgEnd3* plays a pleiotropic role in the response to cell wall integrity agent calcofluor white (CFW), oxidative stress, and fungicide fludioxonil.

## 2. Results

### 2.1. CgEnd3 Is Required for Endocytosis

**Figure 1:**
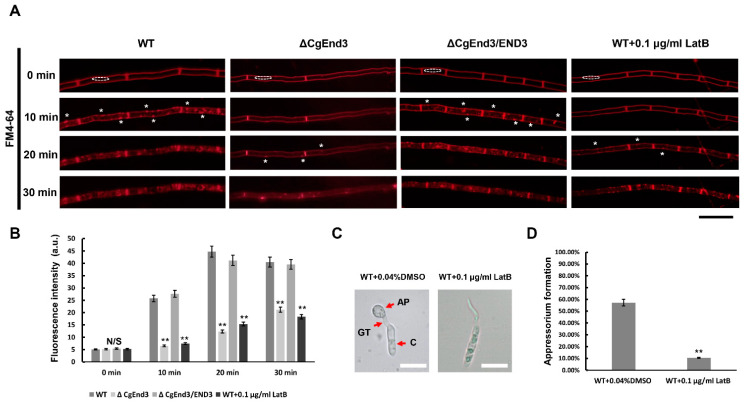
Involvement of *CgEnd3* in endocytosis. (**A**) Hyphal block from wild type (WT), Δ*CgEnd3*, and Δ*CgEnd3*/END3 were inoculated on Potato Dextrose Agar (PDA)-coated glass slides and cultured at 25 °C. At 2 days post inoculation (dpi), hyphae were stained using 0.5 μM N-(3-triethylammoniumpropyl)-4-(p-diethylaminophenylhexatrienyl)-pyridinium 2Br (FM4-64), and images were photographed at different time points (0–30 min) using fluorescence microscope. In any case, hyphae from WT were treated with 0.1 μg/mL Latrunculin B (LatB) for 30 min. The dotted frame indicates the region of cytoplasm, and the fluorescence intensity at each time point was quantified using ImageJ software. The white asterisks indicate site of endocytosis. This experiment was repeated three times. Bars = 10 μm. (**B**) Bar chart showing the mean fluorescence intensity in cytoplasm of each strain at different time points. Data from at least ten hyphae were collected at each time points from each strain. Error bars represent the standard deviations. Data were analyzed using Duncan’s range test. Asterisks ** indicate statistically significant differences at *p* < 0.05. a.u., arbitrary units. (**C**) Conidial suspensions (10^5^ conidia/mL) from WT were inoculated on the hydrophobic side of Gel-bond membrane, water drops were replaced by 30 μL, 0.1 μg/mL LatB at 3 hpi for 30 min, and the controls were treated with 0.04% dimethyl sulfoxide (DMSO). Each sample was washed with distilled water after treatment. Appressorium formation was imaged at 5 h post inoculation (hpi). This experiment was repeated three times. AP = appressorium. GT = germ tube. C = conidia. Bars = 10 μm. (**D**) Bar chart showing the rate of appressorium formation at 5 hpi. Error bars represent the standard deviations. Data were analyzed using Duncan’s range test. Asterisks ** indicate statistically significant differences at *p* < 0.05. N/S = difference not significant.

Endocytosis is a basic cellular process in eukaryotic cells, which internalizes membrane proteins and macromolecules from the extracellular environment. To determine the molecular functions of *CgEnd3* in *C. gloeosporioides*, *CgEnd3* deletion mutants were obtained by replacing the encoding gene with a *Sur* cassette. Subsequently, the full-length sequence of *CgEnd3* was reintroduced into the Δ*CgEnd3* for complementation assay (Figure S2). FM4-64 is a ubiquitous tool in analyzing endocytosis and vesicle trafficking in living fungi and plants. To determine whether *CgEnd3* is required for the normal endocytosis process, the hyphal blocks from WT, Δ*CgEnd3*, and Δ*CgEnd3*/END3 were inoculated on Potato Dextrose Agar (PDA)-coated glass slides. Then, each strain was stained with 5 µM of the lipophilic dye FM4-64 and the internalization of FM4-64 was subsequently observed under a fluorescence microscope. At 0 min, the dye accumulated specifically at the plasma membrane of WT and Δ*CgEnd3*, indicating that the internalization of the dye had not begun. At 10 min, punctate and patchy structures begin to appear at both the plasma membrane and cytoplasm in WT and Δ*CgEnd3*/END3, indicating the rapid uptake of the dye and functional endocytosis process in hyphae. At 20 and 30 min, the dye was dispersed in the whole hyphae of WT and Δ*CgEnd3*/END3. In contrast, only a few dots appeared at the plasma membrane of Δ*CgEnd3* at 20 min, and the internalization of FM4-64 only became obvious at 30 min (Figure 1A). Moreover, the florescence intensity in the cytoplasm of Δ*CgEnd3* was significantly decreased, compared with that of the WT and Δ*CgEnd3*/END3 at different time points (10–30 min) (Figure 1B). To further demonstrate the function of *CgEnd3* in endocytosis, the endocytosis inhibitor, Latruncuin B (LatB), was applied in this study. The results showed that the pattern of FM4-64 internalization was similar between Δ*CgEnd3* and LatB-treated WT (Figure 1A,B). *C. gloeosporioides* formed a specialized infection structure, called appressorium, which plays a vital role in pathogenicity. To determine whether endocytosis is an essential factor for appressorium formation, germinated conidia was exposed to 0.1 μg/mL LatB for 30 min. The results showed that LatB treatment significantly affected the formation of appressorium in WT (Figure 1C,D). Collectively, these results showed that *CgEnd3* played an important role in endocytosis, and normal endocytosis during germination was required for appressorium formation in *C. gloeosporioides.*

### 2.2. Deletion of CgEnd3 Resulted in the Defect of Appressorium Formation, Melanization, Polarity, Penetration and Invasive Growth

**Figure 2:**
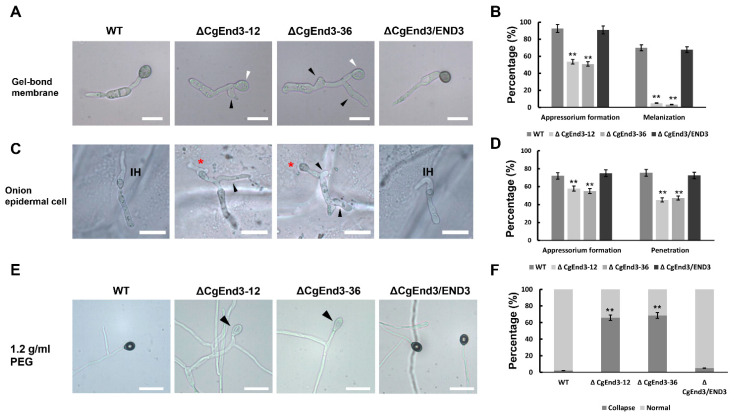
Appressorium formation, penetration and turgor pressure test. (**A**) Equal volumes (30 μL) of conidial suspensions (10^5^ conidia/mL) from WT, Δ*CgEnd3*, and Δ*CgEnd3*/END3 were inoculated on the hydrophobic side of the Gel-bond membrane. Images were pictured at 9 hpi. White arrow indicates the nonmelanized appressorium. This experiment was repeated three times. Black arrow indicates the branched germ tube. Bars = 10 µm. (**B**) Bar chart showing the percentage of appressorium formation and melanization at 9 hpi in (**A**). Error bars represent the standard deviations. Data were analyzed using Duncan’s range test. Asterisks ** indicate a statistically significant differences at *p* < 0.05. (**C**) Appressorium formation and penetration of WT, Δ*CgEnd3*, and Δ*CgEnd3*/END3 on onion epidermal cell at 9 hpi. This experiment was repeated three times. IH = infection hyphae. Red asterisk indicates the stunted infection hyphae. Black arrow indicates the branched germ tube. Bars = 10 µm. (**D**) Bar chart showing the appressorium formation and penetration rate in WT, Δ*CgEnd3*, and Δ*CgEnd3*/END3 on onion epidermal cell at 9 hpi. Error bars represent the standard deviation. Data were analyzed using Duncan’s range test. Asterisks ** indicate statistically significant differences at *p* < 0.05. (**E**) The turgor pressure of appressorium from WT, Δ*CgEnd3*, and Δ*CgEnd3*/END3 were tested using 1.2 g/mL polyethylene glycol (PEG) 4000. Black arrow indicates the collapsed appressorium. This experiment was repeated three times. Bars = 10 µm. (**F**) Bar chart showing the percentage of normal or collapsed appressorium in WT, Δ*CgEnd3*, and Δ*CgEnd3*/END3 under the treatment of PEG 4000 for 10 min. Error bars represent the standard deviations. Data were analyzed using Duncan’s range test. Asterisks ** indicate statistically significant differences at *p* < 0.05.

Since *CgEnd3* regulates endocytosis, and normal endocytosis during germination was required for appressorium formation in *C. gloeosporioides*, we then examined whether or not *CgEnd3* was involved in the development of appressorium. Equal volumes (30 μL) of conidial suspensions (10^5^ conidia/mL) from WT, Δ*CgEnd3*, and Δ*CgEnd3*/END3 were inoculated on the hydrophobic side of the Gel-bond membrane. At 9 hpi, WT and Δ*CgEnd3*/END3 formed numerous appressoria (>90%); over 75% of the appressoria that was formed in WT and Δ*CgEnd3*/END3 were melanized. However, Δ*CgEnd3* showed significantly decreased appressorium formation (≈50%) compared with that of the WT, and only approximately 5% of appressoria were melanized (Figure 2A,B). Moreover, multiple germ tubes were observed in Δ*CgEnd3* (Figure 2A). These results indicate that *CgEnd3* was required for appressorium formation, melanization, and polarity during germination. In previous studies, onion epidermal cells have been extensively used in the analysis of penetration ability in phytopathogenic fungi [48,49]. To test the penetration ability of appressoria in Δ*CgEnd3*, equal volumes (30 μL) of conidial suspensions (10^5^ conidia/mL) from WT, Δ*CgEnd3*, and Δ*CgEnd3*/END3 were inoculated on the onion epidermal cells (hydrophobic surface). The results show that Δ*CgEnd3* displayed similar defects in appressorium formation and polarity during germination, compared with that on the Gel-bond membrane. The penetration rate of appressorium in Δ*CgEnd3* was significantly decreased compared with that of the WT and Δ*CgEnd3*/END3, and Δ*CgEnd3* developed stunted infection hyphae compared with the WT (Figure 2C,D). These results indicated that *CgEnd3* plays an important role in appressorium formation, melanization, polarity, penetration, and invasive growth.

### 2.3. Turgor Pressure Is Decreased in ΔCgEnd3

Melanin, in the appressoria of most plant pathogenic fungi, allows for the buildup of turgor pressure, and turgor pressure provides the essential driving force that enables the fungal peg to rupture the host cuticle [16]. In this study, the melanization of appressoria in Δ*CgEnd3* was impaired significantly, and the penetration rate of Δ*CgEnd3* was also decreased. To determine whether turgor pressure in Δ*CgEnd3* was decreased, turgor pressure was tested using a high concentration of PEG 4000 (1.2 g/mL). Conidial suspensions (10^5^ conidia/mL) from WT, Δ*CgEnd3*, and Δ*CgEnd3*/END3 were inoculated on the hydrophobic side of the Gel-bond membrane. At 12 hpi, the water drop was replaced by 30 µL 1.2 g/mL of PEG 4000. After 10 min of treatment, the collapse rate of appressorium in Δ*CgEnd3* was significantly higher than that of the WT and Δ*CgEnd3*/END3 (Figure 2E,F). This result indicates that the turgor pressure in Δ*CgEnd3* was significantly decreased, which further affected the penetration ability of appressoria.

### 2.4. ΔCgEnd3 Loses the Ability of Cellophane Membrane Penetration

**Figure 3:**
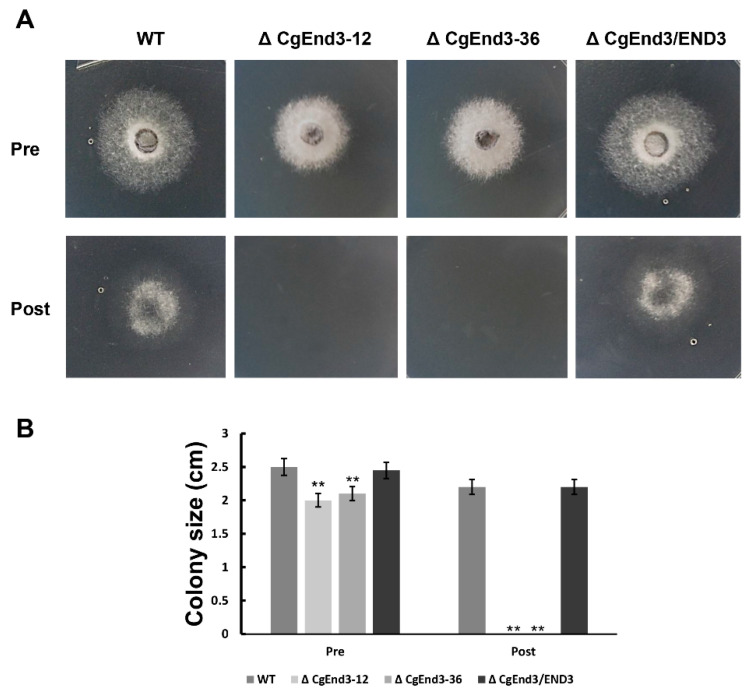
Cellophane membrane penetration assay. (**A**) Hyphal blocks from WT, Δ*CgEnd3*, and Δ*CgEnd3*/END3 were inoculated on cellophane membranes overlaid on PDA medium for 2 days at 25 °C (Pre). The cellophane membrane were removed, and the resulting plates were incubated at 25 °C for 2 additional days (Post). This experiment was repeated three times. (**B**) Bar chart showing the colony size of each strain at 2 days post removal of cellophane membrane (Post). Error bars represent the standard deviations. Data were analyzed using Duncan’s range test. Asterisks ** indicate statistically significant differences at *p* < 0.05.

Cellophane is a permeable membrane, which is widely used in the penetration test of pathogenic fungi [50,51,52]. To determine the penetration ability of Δ*CgEnd3*, the hyphal blocks from WT, Δ*CgEnd3*, and Δ*CgEnd3*/END3 were inoculated on the sterile cellophane membrane overlaid on PDA plates. Plates was cultured at 25 °C for 2 days (Pre). At 2 dpi, the entire membrane with colony was removed, and the resulting plates were cultured at 25 °C for 2 days (Post). The results show that the colonies of WT and Δ*CgEnd3*/END3 were developed on PDA plates after the removal of cellophane membrane, indicating that WT and Δ*CgEnd3*/END3 successfully penetrated the cellophane membrane, but Δ*CgEnd3* did not (Figure 3). Prolonged incubation before the removal of the cellophane membrane also failed to observe the successful penetration of Δ*CgEnd3* (data not shown). This result indicated that Δ*CgEnd3* lost its cellophane membrane penetration ability.

### 2.5. CgEnd3 Is Involved in Oxidant Adaptation and the Regulation of Effectors during Early Infection Stage

**Figure 4:**
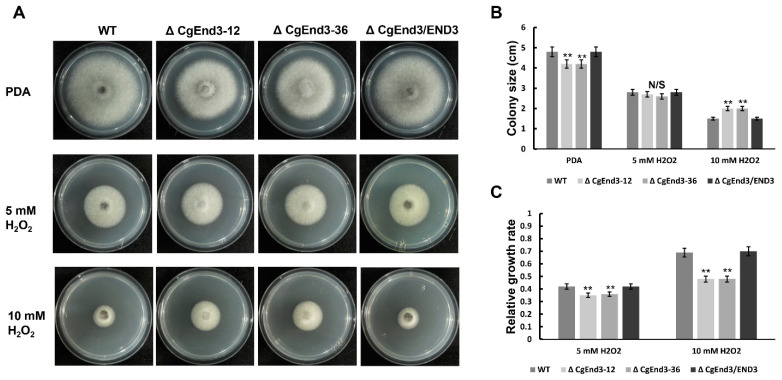
Vegetative growth under oxidative stress. (**A**) Hyphal block from WT, Δ*CgEnd3*, and Δ*CgEnd3*/END3 were inoculated on PDA or PDA mixed with 5 and 10 mM H_2_O_2_, respectively. Images were pictured at 4 dpi. This experiment was repeated three times. (**B**) Bar chart showing the colony size of each strain on PDA and PDA mixed with 5 and 10 mM H_2_O_2_. Error bars represent the standard deviations. Data were analyzed using Duncan’s range test. Asterisks ** indicate statistically significant differences at *p* < 0.05. N/S = difference not significant. (**C**) Bar chart showing the relative growth rate of each strain on PDA mixed with 5 and 10 mM H_2_O_2_; computing methods of relative growth rate were described by Wang et al. [54]. The deceased relative growth rate indicates the increased resistance to stress and vice versa. Error bars represent the standard deviations. Data were analyzed using Duncan’s range test. Asterisks ** indicate statistically significant differences at *p* < 0.05.

**Figure 5:**
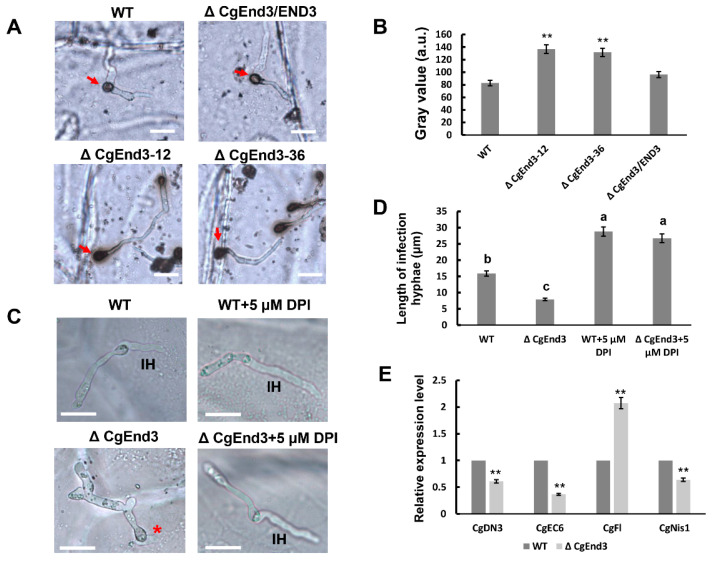
*CgEnd3* is involved in oxidant adaptation and expression of core effectors during the penetration of appressorium. (**A**) Equal volumes (30 μL) of conidial suspensions (10^5^ conidia/mL) from WT, Δ*CgEnd3,* and Δ*CgEnd3*/END3 were inoculated on the hydrophobic side of onion epidermal cell. At 9 hpi, appressorium of each strain was stained using 2 mg/mL 3,3′-diaminobenzidine (DAB) solution in darkness for 12 h. Red arrow indicates the dark brown polymers in the presence of reactive oxygen species (ROS). This experiment was repeated three times. Bars = 10 µm. (**B**) The deposition of dark brown polymers was analyzed using ImageJ software, bar chart showing the gray value (a. u.) of each strain. Data were collected from at least 10 samples from each strain. Error bars represent the standard deviations. Data were analyzed using Duncan’s range test. Asterisks ** indicate statistically significant differences at *p* < 0.05. (**C**) Conidial suspensions (10^5^ conidia/mL) from WT and Δ*CgEnd3* were added with diphenyleneiodonium (DPI) at a final concentration of 5 μM. Images were photographed at 9 hpi. Red asterisk indicates the stunted infection hyphae. IH = infection hyphae. Bars = 10 µm. (**D**) Bar chart showing the mean length of infection hyphae from each sample. Data were collected from at least 20 infection hyphae, and this experiment was repeated three times. Error bars represent the standard deviations. The values indicated by the different letters are significantly different at *p* < 0.05, as determined using post hoc Tukey’s test. (**E**) Conidial suspensions (10^6^ conidia/mL) from WT and Δ*CgEnd3* were inoculated on poplar leaves. Inoculation areas (including fungal tissue and leaf tissue) were cut at 3 dpi, and 5 mg samples from WT and Δ*CgEnd3* areas were collected. Bar chart showing the expression of conserved effectors in WT and Δ*CgEnd3* during early stage of infection. This experiment was repeated three times. Error bars represent the standard deviations. Data were analyzed using Duncan’s range test. Asterisks ** indicate statistically significant differences at *p* < 0.05.

In plants, reactive oxygen species (ROS) plays a vital role in the defense against phytopathogens, and active oxygen bursts are among the earliest responses at the infection site [53]. Thus, oxidant adaptation is crucial for the successful infection of plant pathogenic fungi. To test the role of *CgEnd3* in the response to oxidative stress, the hyphal blocks of WT, Δ*CgEnd3*, and Δ*CgEnd3*/END3 were inoculated on PDA and PDA containing 5 or 10 mM H_2_O_2_, respectively. At 4 dpi, the colony sizes of WT, Δ*CgEnd3*, and Δ*CgEnd3*/END3 were recorded, and the relative growth rate was calculated. The results show that the deletion of *CgEnd3* resulted in the defects in vegetative growth on PDA, but Δ*CgEnd3* displayed resistance to oxidative stress compared with that of the WT and Δ*CgEnd3*/END3, especially when under the treatment of 10 mM H_2_O_2_ (Figure 4), suggesting that *CgEnd3* plays a negative role in oxidative stress response. To determine whether the deletion of *CgEnd3* affected the ROS adaptation during penetration, conidial suspensions (10^5^ conidia/mL) from WT, Δ*CgEnd3*, and Δ*CgEnd3*/END3 were inoculated on the onion epidermal cell. At 9 hpi, water drops of each strain were replaced with 2 mg/mL 3,3′-diaminobenzidine (DAB) solution, and each sample was incubated in the dark for 12 h. The results show that the ROS accumulated around the appressoria with dark brown polymers (Figure 5A), and the degree of staining for the appressorium of the Δ*CgEnd3* was greater than that observed in WT and Δ*CgEnd3*/END3 (Figure 5A,B), indicating that the deletion of *CgEnd3* resulted in oxidant adaptation defects during penetration. To determine whether the stunted infection hyphae in Δ*CgEnd3* was caused by the defect of oxidant adaptation during penetration, the ROS inhibitor, diphenyleneiodonium (DPI), was added during conidial germination at a final concentration of 5 μM, and the result shows that the stunted infection hyphae in Δ*CgEnd3* was successfully rescued by exogenously added DPI (Figure 5C,D). These results indicate that *CgEnd3* is responsible for oxidant adaptation during penetration, which affects invasive growth.

Fungal effectors are secreted by phytopathogens to manipulate plant immunity. Moreover, certain effectors are conserved among different pathogens and regarded as core effectors; therefore, they may play an important and common role in pathogen virulence. In this study, the deletion of *CgEnd3* resulted in severe defects in penetration, invasive growth, and oxidant adaptation, which indicates that the mutant is defective in host immune suppression and may be connected to the expression of core effectors. In this study, we identified effector genes or effector candidates that are homologous to other *Colletotrichum* species and suppress host immunity and facilitate invasive growth during the early infection stage, including CgDN3 (protein ID: 1717571) in *C. gloeosporioides* [55]; CgEC6 (protein ID: 1730105), which shares homology (82.1%) to *C. higginsianum* ChEC6 [56]; CgFl (protein ID: 1731882), which shares homology (82.3%) to *Colletotrichum graminicola* CgFl [57]; and CgNis1 (protein ID: 31327), which shares homology (68.5%) to *C. orbiculare* CoNis1 [58]. In addition, our team previously performed a histopathology study of poplar leaves infected by *C. gloeosporioides*, and the results showed that the infection peg formed at the base of the appressorium expanded to form an infection vesicle after penetrating the host cuticle and epidermal cell wall at 3 dpi [6]. Therefore, the total RNA of the WT and Δ*CgEnd3* during the early infection stage on poplar leaves at 3 dpi were extracted, and qRT showed that the expression level of *CgDN3*, *CgEC6,* and *CgNis1* were significantly decreased in Δ*CgEnd3*, and the expression of *CgFl* was significantly upregulated in Δ*CgEnd3* compared with that of the WT (Figure 5E). This result indicates that is *CgEnd3* involved in the regulation of core effectors during the early infection stage. Collectively, these results show that a lack of *CgEnd3* resulted in decreased ability in the suppression of host immunity.

### 2.6. CgEnd3 Is Required for the Full Virulence

**Figure 6:**
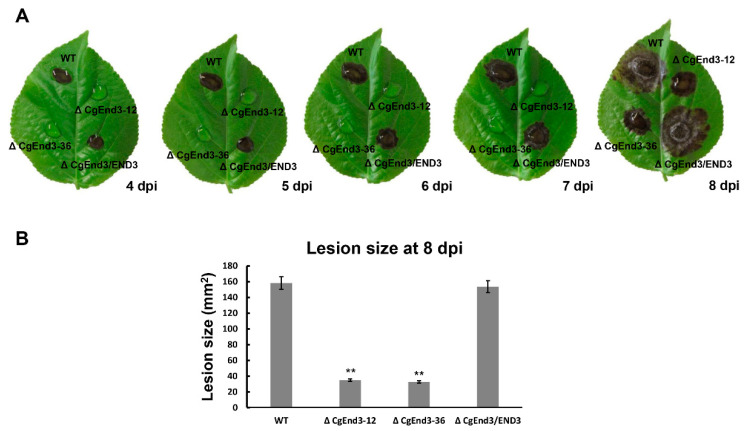
Pathogenicity assay on poplar leaves. (**A**) Equal volumes (30 μL) of conidial suspensions (2 × 10^5^ conidia/mL) from WT, Δ*CgEnd3*, and Δ*CgEnd3*/END3 were inoculated on poplar leaves. Then, leaves were cultured at 25 °C under moist environment. Images were pictured at 4–8 dpi. This experiment was repeated three times. (**B**) Bar chart showing the lesion sizes of WT, Δ*CgEnd3*, and Δ*CgEnd3*/END3 at 8 dpi. Error bars represent the standard deviations. Data were analyzed using Duncan’s range test. Asterisks ** indicate statistically significant differences at *p* < 0.05.

In *C. gloeosporioides*, there are strong correlations between appressoria and pathogenicity. Additionally, it is clear that a lack of *CgEnd3* resulted in pleiotropic defects in appressorium formation, penetration, invasive growth, oxidant adaptation, and the expression of core effectors. Therefore, we further determined the role of *CgEnd3* in the pathogenicity of *C. gloeosporioides*. Equal volumes (30 μL) of conidial suspensions (2 × 10^5^ conidia/mL) from WT, Δ*CgEnd3*, and Δ*CgEnd3*/END3 were inoculated on poplar leaves. At 4 dpi, necrotic lesions appeared in the inoculation area of WT and Δ*CgEnd3*/END3, and the lesions gradually expanded between 4 and 8 dpi. However, no necrotic lesions were shown in Δ*CgEnd3* at 4–7 dpi (Figure 6A). Since the Δ*CgEnd3* was able to form appressoria on the Gel-bond membrane and onion epidermal cell, we presumed that the Δ*CgEnd3* may retain weak pathogenicity, and prolonged observation (8 dpi) demonstrated that the pathogenicity in Δ*CgEnd3* was significantly decreased but not fully abrogated (Figure 6A,B). These results indicate that *CgEnd3* was required for full virulence, and the significantly reduced virulence of Δ*CgEnd3* was caused by the decreased ability of appressorium formation, penetration, and invasive growth, which is also attributed to decreased ability in suppressing host immunity.

### 2.7. CgEnd3 Involved in the Calcium Signaling in C. gloeosporioides

**Figure 7:**
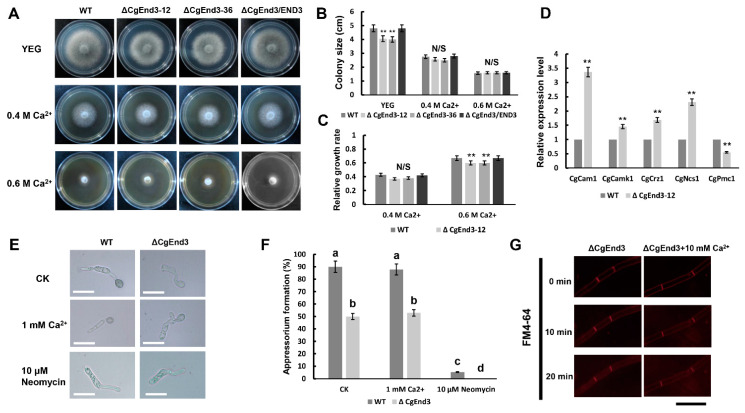
Role of CgEnd3 in calcium signaling. (**A**) Hyphal block from WT, Δ*CgEnd3*, and Δ*CgEnd3*/END3 were inoculated on Yeast extract-glucose medium (YEG) and YEG containing 0.4 and 0.6 M Ca^2+^, respectively. Strains were cultured at 25 °C for 4 days. This experiment was repeated three times. (**B**) Bar chart showing the colony size of each strain on YEG and YEG containing 0.4 and 0.6 M Ca^2+^ in (**A**). Data were analyzed using Duncan’s range test. Asterisks ** indicate statistically significant differences at *p* < 0.05. N/S = difference not significant. (**C**) Bar chart showing the relative growth rate of each strain under the treatment of 0.4 and 0.6 M Ca^2+^. Error bars represent the standard deviations. Data were analyzed using Duncan’s range test. Asterisks ** indicate statistically significant differences at *p* < 0.05. N/S = difference not significant. (**D**) Bar chart showing the relative expression of five calcium signaling genes in WT and Δ*CgEnd3*. This experiment was repeated three times. Error bars represent the standard deviations. Data were analyzed using Duncan’s range test. Asterisks ** indicate statistically significant differences at *p* < 0.05. (**E**) The effects of exogenous 1 mM Ca^2+^ and 10 μM of the phospholipase C inhibitor neomycin on appressorium formation in WT and Δ*CgEnd3* at 9 hpi. This experiment was repeated three times. Bars = 10 µm. (**F**) Bar chart showing the rate of appressorium formation under the treatment of 1 mM Ca^2+^ or 10 μM neomycin. Error bars represent the standard deviations. The values indicated by the different letters are significantly different at *p* < 0.05, as determined using post hoc Tukey’s test. (**G**) The internalization of FM4-64 in Δ*CgEnd3* in the presence of 10 mM Ca^2+^. Images were photographed at different time points (0–20 min) using fluorescence microscope. Bars = 10 μm. This experiment was repeated three times.

CgEnd3 encodes a predicted 397 amino acid protein and contains four calcium ion-binding sites (Figure S1A), indicating that CgEnd3 may have a role in calcium signaling. To determine whether *CgEnd3* is involved in calcium signaling, hyphal blocks from WT, Δ*CgEnd3*, and Δ*CgEnd3*/END3 were inoculated on the Yeast extract-glucose medium (YEG) and YEG containing 0.4 M and 0.6 M Ca^2+^, respectively. The results show that the deletion of *CgEnd3* also caused defects in vegetative growth on YEG and significantly increased the resistance to 0.6 M Ca^2+^ stress (Figure 7A–C), indicating that *CgEnd3* plays a negative role in the response to Ca^2+^ stress. To further illuminate the role of *CgEnd3* in calcium signaling, the transcription level of various calcium signaling genes, including calcineurin-responsive zinc finger transcription factor CgCrz1 (protein ID: 1748597), calmodulin CgCam1 (protein ID: 1748141), calcium transporting ATPase CgPmc1 (protein ID: 1729318), Ca^2+^ sensor CgNcs1 (protein ID: 50723), and Ca^2+^/calmodulin-dependent protein kinase CgCamk1 (protein ID: 1743859), were tested using qRT-PCR analysis. The results show that the deletion of *CgEnd3* leads to significantly increased transcription levels of *CgCrz1*, *CgCam1*, *CgNcs1*, and *CgCamk1*, but it decreased the transcription level of *CgPmc1* (Figure 7D). Interestingly, CgCam1, CgNcs1, CgCamk1, and CgEnd3 all possess the EF hand domain or belong to the EF hand protein superfamily, and previous studies have revealed that the EF hand protein superfamily is composed of a large number of functionally diverse Ca^2+^ binding proteins, and they play vital roles in Ca^2+^ homeostasis and Ca^2+^ signaling mechanisms [59,60], indicating that *CgEnd3* may have a negative regulation toward the transcription of the EF hand superfamily. Additionally, there exists the possibility that the upregulated expression of EF hand proteins was caused by a genetic compensation due to the deletion of *CgEnd3*. Collectively, these results suggested that *CgEnd3* plays an important role in calcium signaling in *C. gloeosporioides*.

Calcium signaling has a conserved function in the regulation of appressorium formation in *M. oryzae* [61], and calcium channel blockers specifically reduced appressorium formation in *C. gloeosporioides* [62]. Therefore, we firstly tested the effect of calcium signaling inhibitor neomycin in *C. gloeosporioides*, and we found that the exogenous supplement of neomycin specifically inhibited appressorium formation in WT and Δ*CgEnd3* without affecting conidial germination (Figure 7E,F). This proved that calcium signaling is involved in the regulation of appressorium formation in *C. gloeosporioides*. To determine whether the defect of appressorium formation in Δ*CgEnd3* has a connection with its role in calcium signaling, the effect of the exogenous addition of Ca^2+^ on appressorium formation was tested. This demonstrated that the exogenous addition of 1 mM Ca^2+^ failed to rescue the defects of appressorium formation in Δ*CgEnd3* (Figure 7E,F). Other concentrations of exogenous Ca^2+^ also demonstrated no replenishment effect (data not shown). In addition, another core function of *CgEnd3* is to regulate normal endocytosis. Therefore, we intended to explore whether the endocytosis regulated by *CgEnd3* is related to the calcium signal, and the results show that exogenous Ca^2+^ also failed to rescue the endocytosis defect in Δ*CgEnd3* (Figure 7G). Collectively, these results indicated that *CgEnd3* regulated appressorium formation and endocytosis in a calcium signaling-independent manner.

### 2.8. CgEnd3 Is Required for Cell Wall Integrity

**Figure 8:**
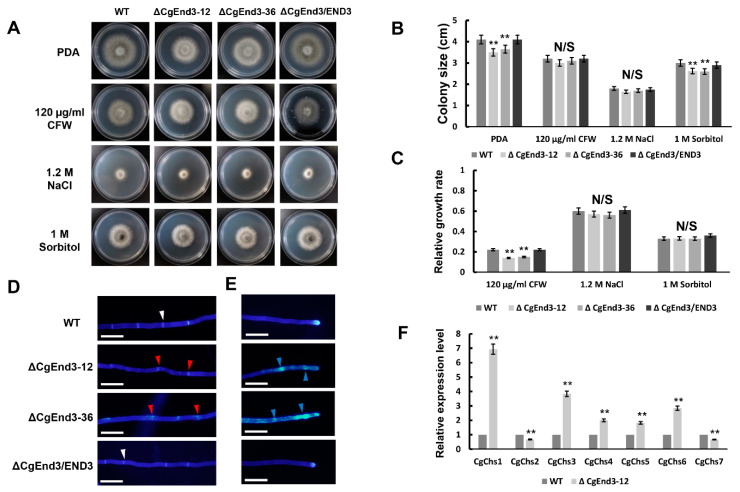
Response to cell wall integrity agents and high osmotic stress, calcofluor white (CFW) staining of hyphae and relative expression of seven chitin synthase genes. (**A**) Vegetative growth of WT, Δ*CgEnd3*, and Δ*CgEnd3*/END3 on PDA and PDA containing 120 µg/mL CFW, 1.2 M NaCl, and 1 M sorbitol, respectively. Strains were cultured at 25 °C for 4 days. This experiment was repeated three times. (**B**) Bar chart showing the colony size of each strain in (**A**). Error bars represent the standard deviations. Data were analyzed using Duncan’s range test. Asterisks ** indicate statistically significant differences at *p* < 0.05. N/S = difference not significant. (**C**) Bar chart showing the relative growth rate of each strain in the presence of 120 µg/mL CFW, 1.2 M NaCl, and 1 M sorbitol, respectively. Error bars represent the standard deviations. Data were analyzed using Duncan’s range test. Asterisks ** indicate statistically significant differences at *p* < 0.05. N/S = difference not significant. (**D**) CFW staining of hyphal septa from WT, Δ*CgEnd3*, and Δ*CgEnd3*/END3. White arrow indicates the intact septum in WT. Red arrow indicates the dispersed chitin at septum. Bars = 10 µm. (**E**) CFW staining of hyphal tip from WT, Δ*CgEnd3*, and Δ*CgEnd3*/END3. Blue arrow indicates the punctiform pattern of chitin distribution at the hyphal tips. This experiment was repeated three times. Bars = 10 µm. (**F**) Bar chart showing the relative expression level of seven chitin synthase genes in WT and Δ*CgEnd3*. This experiment was repeated three times. Data were analyzed using Duncan’s range test. Asterisks ** indicate statistically significant differences at *p* < 0.05.

Calcium signaling has a conserved connection with cell-wall integrity (CWI) signaling in various fungi [34]. To determine the function of *CgEnd3* in abiotic stress response, hyphal blocks from WT, Δ*CgEnd3*, and Δ*CgEnd3*/END3 were inoculated on PDA containing 1.2 M NaCl, 1 M sorbitol, and 120 µg/mL CFW, respectively. The results indicate that Δ*CgEnd3* showed similar restriction rates in the presence of 1.2 M NaCl and 1 M sorbitol, compared with those of the WT and Δ*CgEnd3*/END3 (Figure 8A–C), indicating that *CgEnd3* was dispensable for the response to osmotic stress. However, Δ*CgEnd3* showed resistance to 120 µg/mL cell wall integrity agent CFW compared with that of the WT and Δ*CgEnd3*/END3 (Figure 8A–C). This result indicated that *CgEnd3* plays a role in cell-wall integrity. The cell wall of filamentous fungi is the place of first contact with external stresses and is mainly composed of chitin [63]. *S. cerevisiae End3p* is required for the correct distribution of chitin at the cell surface [47]. In this study, CFW staining was used to investigate the deposition of chitin in hyphae. Hyphae from each strain were stained using 1 μg/mL CFW for 3 min under darkness. In WT, chitin was mainly located in the septum and hyphal tip, and it was equally distributed on the cell wall. In contrast, chitin at septum in Δ*CgEnd3* was dispersed, which represents fragmentary septum. In addition, chitin was deposited in a punctiform pattern at the hyphal tips (Figure 8D,E). In filamentous fungi, the chitin synthase was reported to be responsible for the synthesis of chitin [64]. In *S. cerevisiae* [65], *Metarhizium acridum* [66], *Fusarium graminearum* [67], and *B. cinerea* [68] chitin synthase are required for cell-wall integrity. In this study, we identified seven chitin synthases CgChs1–CgChs7 (Protein IDs: 1900258, 361644, 1836492, 1744167, 1830816, 1462050, and 30251, respectively) in *C. gloeosporioides*, and qRT-PCR was applied to determine the relative expression levels of these genes in WT and Δ*CgEnd3*. The results show that the transcription levels of *CgChs1*, *CgChs3*, *CgChs4*, *CgChs5,* and *CgChs6* were significantly increased in Δ*CgEnd3* compared with those of the WT, while *CgChs2* and *CgChs7* were significantly decreased (Figure 8F). These results indicate that *CgEnd3* plays an important role in the cell-wall integrity of *C. gloeosporioides.*

### 2.9. Lack of CgEnd3 Resulted in Increased Resistance to Fungicide Fludioxonil

**Figure 9:**
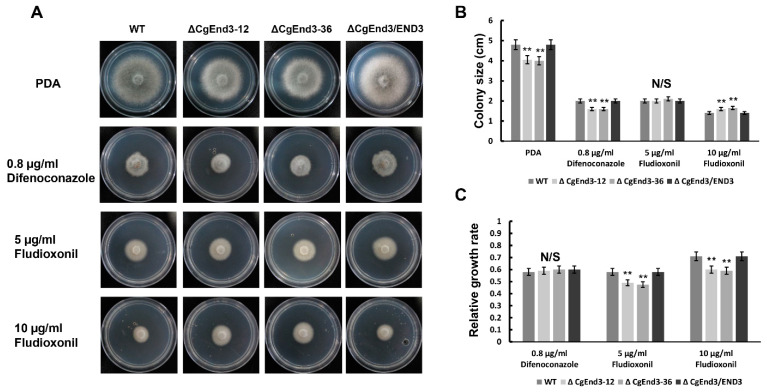
Response to the fungicide fludioxonil. (**A**) Vegetative growth of WT, Δ*CgEnd3*, and Δ*CgEnd3*/END3 on PDA and PDA containing 0.8 µg/mL difenoconazole, 5 and 10 µg/mL fludioxonil, respectively. Strains were cultured at 25 °C for 4 days. This experiment was repeated three times. (**B**) Bar chart showing the colony size of each strain in (**A**). Data were analyzed using Duncan’s range test. Asterisks ** indicate statistically significant differences at *p* < 0.05. N/S = difference not significant. (**C**) Bar chart showing the relative growth rate of each strain on PDA containing 0.8 µg/mL difenoconazole, 5 and 10 µg/mL fludioxonil, respectively. Data were analyzed using Duncan’s range test. Asterisks ** indicate statistically significant differences at *p* < 0.05. N/S = difference not significant.

Previous studies also illustrated a connection between calcium signaling and drug resistance in pathogenic fungus [69]. To determine the role of *CgEnd3* in response to antifungal drugs, two types of broad-spectrum fungicides of anthracnose, difenoconazole and fludioxonil [70], were tested. Hyphal blocks from WT, Δ*CgEnd3,* and Δ*CgEnd3*/END3 were inoculated on PDA containing 0.8 µg/mL difenoconazole and 5 and 10 µg/mL fludioxonil, respectively. At 4 dpi, each strain was significantly suppressed, but the relative growth rate of Δ*CgEnd3* was similar with that of the WT under the treatment of 0.8 µg/mL difenoconazole. However, Δ*CgEnd3* showed significantly increased resistance to 5 and 10 µg/mL fludioxonil compared with that of the WT (Figure 9). This result showed that *CgEnd3* plays a negative role in the resistance to fludioxonil.

## 3. Discussion

In this study, we characterized the CgEnd3 homologous to the EH domain-containing protein End3p of *S. cerevisiae.* In *S. cerevisiae*, End3p cooperated with another EH domain-containing protein Pan1p to regulate the cortical actin cytoskeleton and endocytosis [45]. Further study revealed that Pan1p and End3p mixes interact with another protein, Sla1p, which is also known to be required for the assembly of cortical actin. The Pan1p–End3p–Sla1p complex is required for actin cytoskeleton and cell-wall morphogenesis [71], and it links Arp2/3-mediated actin assembly to sites of clathrin-mediated endocytosis [46]. These studies indicated that End3p plays important roles in the actin cytoskeleton, cell wall morphogenesis, and endocytosis. In *M. oryzae*, *MoEnd3* plays an important role in endocytosis, and the deletion of *MoEnd3* affected appressorium formation and pathogenicity [72]. In this study, the deletion of *CgEnd3* also resulted in defects of endocytosis, indicating that End3 may act as a conserved regulator of endocytosis in fungi. Moreover, a lack of *CgEnd3* resulted in decreased vegetative growth and deficient polarity during germination. In previous studies, it was found that filamentous fungi are able to spread to the hyphae at a speed of 18.5 μm/min, and the rapid spreading of hyphae requires normal endocytosis [73]. Both exo- and endocytosis were reported to be involved in polarized growth in *U. maydis* and *S. cerevisiae* [74,75]. Therefore, *CgEnd3* may regulate vegetative growth and polarity in an endocytosis-dependent way. Aside from the perspectives of growth and polarity, *CgEnd3* also acts as a key regulator in pathogenicity-related functions, including appressorium formation, appressorium melanization, turgor pressure, penetration, and invasive growth. The occurrence of these phenotypes is valid, because endocytosis is the basic physiological process of cells, pleiotropic defects will occur when endocytosis is blocked, and endocytosis is required for appressorium formation in *C. gloeosporioides* (Figure 1C,D). In any case, the underlying connection between endocytosis and appressorium formation was supported by the study of *MoEnd3*, since the deletion of *MoEnd3* showed severe defects in endocytosis and the endocytosis-mediated internalization of cell membrane sensors, MoSho1 and MoPth11 [72]. In many fungi, Sho1 and Pth11 were reported as upstream sensors in the Pmk1 mitogen-activated protein (MAP) kinases (MAPK) and cAMP pathways to regulate host signal recognition and appressorium formation, respectively [9,10,11,76]. Therefore, the defects of appressorium formation in Δ*CgEnd3* may be due to the defects in endocytosis-mediated internalization of cell membrane sensors in *C. gloeosporioides.* Further experiments are needed to illuminate the underlying mechanisms of endocytosis-dependent appressorium formation.

In fungi, the cell wall protects cells from the harm of environmental stresses, including temperature, pH, and lysing enzyme. CWI signaling plays an important role in maintaining the morphology and functions of the cell wall, and the CWI pathway helps fungi to reinforce the cell wall [77]. In *S. cerevisiae*, the molecular mechanism of CWI is well understood. There are five mitogen-activated protein (MAP) kinase pathways in yeast, and the Slt2 MAP kinase pathway plays a central role in CWI. The Slt2 MAP kinases consist of MAPKKK Bck1, MAPKK Mkk1, and MAPK Slt2, and there are several downstream transcription factors, including Swi4, Swi6, and Rlm1, which are also involved in the regulation of CWI [77,78]. In filamentous fungi, several genes that are required for CWI have also been identified. In *M*. *oryzae*, the deletion of *Mps1* (Slt2 ortholog) resulted in defects in aerial hyphae growth and conidiation, appressorium penetration, and pathogenicity [79,80]. In *C. gloeosporioides*, the deletion of *CgSlt2* resulted in the defects in appressorium formation, sporulation, polarized growth, and pathogenicity. However, the function of *CgSlt2* in CWI is not yet clear, but the spores harvested from the *CgSlt2* mutant cultured on medium mixed with sorbitol developed small and nonmelanized appressorium-like structures [81]. In addition, two small G proteins, CgRhoB and CgCdc42, are involved in CWI, and their deletion affected the responses to cell-wall stress agents and protoplast release. In any case, Δ*CgRhoB* and Δ*CgCdc42* showed an abnormal distribution of chitin in hyphae, and *CgCdc42* is involved in the regulation of chitin synthase genes [3,54]. The putative upstream cell surface sensor, CgSho1, also plays an important role in cell-wall integrity [82]. In this study, the deletion of *CgEnd3* affected the transcription of chitin synthase genes *CgChs1*–*CgChs7*, and it affected the response to the cell-wall integrity stress agent. Additionally, the chitin distribution at the septum and hyphal tip was also changed, compared to that of the WT. These results indicated that *CgEnd3* plays an important role in CWI. Moreover, CgEnd3 contains four calcium ion binding sites, and it is involved in calcium signaling in *C. gloeosporioides.* Previous reports reveal that calcineurin plays an important role in the virulence pathways of eukaryotic microbial pathogens, including invasive growth, drug tolerance, and CWI. In *Sclerotinia sclerotiorum*, calcineurin signaling is involved in CWI [34]. In *V. dahliae*, the deletion of calcineurin-responsive zinc finger transcription factor *VdCrz1* showed hypersensitivity to the cell-wall perturbing agent, SDS [36]. In *B. cinerea*, the deletion of *BcCrz1* also caused defects in CWI [37]. In *M. oryzae*, phospholipase C (PLC) acts as a Ca^2+^ supplier by generating Ca^2+^-releasing molecules, and two phospholipase C genes, *MoPlc2* and *MoPlc3*, were involved in CWI. The deletion of *MoPlc2* and *MoPlc3* affected the response to CWI stress agent CFW and showed hypersensitivity to the lysing enzyme [83]. In *Candida albicans*, Ca^2+^/H^+^ antiporter GaGdt1 and calcium pump CaPmr1 have cooperative roles in CWI [84]. Collectively, those studies indicated that calcium signaling possesses a conserved connection with CWI, and the involvement of *CgEnd3* in calcium signaling may further affect CWI in *C. gloeosporioides.*

In this study, the deletion of *CgEnd3* resulted in increased resistance to the cell wall stress agent CFW, oxidative stress, calcium ion stress, and fungicide fludioxonil, indicating that *CgEnd3* acts as a negative regulator in response to multiple stresses. In *C. gloeosporioides*, the mitogen-activated protein kinase, CgHog1, is the central regulator for the resistance to fludioxonil, and the deletion of *CgHog1* showed enhanced resistance to fludioxonil. In any case, the analysis of genome-wide transcription patterns in *C. gloeosporioides* showed that Ca^2+^-transporting ATPase was downregulated under the treatment of 10 μg/mL of fludioxonil, indicating the involvement of calcium signaling in drug resistance [70]. Previous reports also suggested that calcineurin is required for virulence and drug resistance in a diverse group of fungi, mainly in human fungal pathogens, including azole tolerance in *C. albicans* [85], fluconazole tolerance in *Cryptococcus gattii* [86], and azole and echinocandin resistance in *Aspergillus fumigatus* [87]. Therefore, it is intriguing to further identify the underlying mechanisms of the relationship between calcium signaling and drug resistance.

In *C. gloeosporioides* and other appressorium-forming fungi, there are strong connections between appressoria and virulence [5,52]. In this study, the deletion of *CgEnd3* showed pleiotropic defects in appressorium-related functions, including appressorium formation, appressorium melanization, turgor pressure, and penetration. In plants, ROS plays a crucial role in the defense against infections by pathogens, and oxidative bursts are among the earliest responses at the infection site [53]. Therefore, oxidant adaptation is also crucial for the infection of plant pathogenic fungi. In *C. gloeosporioides*, we previously revealed the significance of ROS tolerance to pathogenicity. The deletion of the zinc finger transcription factor gene, *CgAp1*, leads to defects in ROS tolerance and reduced pathogenicity [4]. The small G protein CgRhoB is involved in oxidative stress response and ROS tolerance during penetration. The deletion of *CgRhoB* resulted in reduced pathogenicity [3]. The deletion of another small G protein *CgCdc42* also showed defects in ROS tolerance, and it further impacted the pathogenicity. Moreover, the exogenous treatment of ROS inhibitor diphenyleneiodonium (DPI) rescued the pathogenicity of the *CgCdc42* deletion mutant [54]. These results showed that both appressorium- and ROS-related functions are vital factors in the pathogenicity of *C. gloeosporioides.* In this study, *CgEnd3* plays an important role in pathogenicity, and the reduced pathogenicity may be caused by the multiple defects of Δ*CgEnd3* in appressorium formation and penetration-related functions. Intriguingly, the defect of invasive growth was rescued by the exogenous addition of DPI, and the transcription of core effectors was regulated by *CgEnd3* during the early infection stage. These results indicated the participation of *CgEnd3* in the suppression of host immunity. In addition, the penetration of the cellophane membrane was abolished in Δ*CgEnd3*, indicating that there are connections between the ability of penetration and pathogenicity. In other pathogenic fungi, including *M. oryzae* [88], *F. oxysporum* [89], *V. dahliae* [90], and *Colletotrichum* species [52], cellophane membrane penetration is an important indicator for their pathogenicity. However, no reports revealed the mechanism of the connection between the penetration of cellophane membrane and pathogenicity. Therefore, we observed the interface between cellophane membrane and PDA plates, but no obvious penetration structures or appressoria were formed at the interface (data not shown), indicating that *C. gloeosporioides* may penetrate the cellophane membrane through hyphae or unknown microstructure that are differentiated from hyphae.

Collectively, our study determined the key role of *CgEnd3* in regulating endocytosis and revealed the novel function of *End3* in calcium signaling. Moreover, *CgEnd3* plays important roles in appressorium formation, appressorium melanization, turgor pressure accumulation, penetration, invasive growth, expression of core effectors, and pathogenicity. The deletion of *CgEnd3* showed negative responses to the cell-wall integrity agent CFW, calcium ion stress, oxidative stress, and fungicide fludioxonil. Our results revealed the pleiotropic roles of *CgEnd3* in *C. gloeosporioides* and suggested that *CgEnd3* or endocytosis-related genes function as promising antifungal targets.

## 4. Materials and Methods

### 4.1. Cultivation of Fungi

The *Colletotrichum gloeosporioides* sensu stricto (s.s.) CFCC80308 strain was isolated from the leaves of *Populus × beijingensis* with symptoms of anthracnose in Beijing Botanical Garden, conserved in the China Forestry Culture Collection Center (CFCC), and supplied by the Key Laboratory for Silviculture and Conservation of Ministry of Education, Beijing Forestry University (Beijing, China) was served as the WT throughout this study [2,3]. WT, and other derivative strains, were maintained on solid PDA medium at 25 °C. Liquid complete medium (1 L CM, 50 mL 20 × nitrate salts, 1 mL vitamin solution, 1 mL 1000 × Trace, 10 g glucose, 2 g peptone, 1 g yeast extract, 1 g casamino acids) was applied to generate the shaking culture of strains. For the selection of sulfonylurea resistant strains, TB3 medium (1 L TB3, 3 g yeast extract, 3 g casamino acids, 200 g sucrose, and 7 g agar) was used to screen the deletion mutants. For the fluorescence microscopy of CFW staining, conidial suspensions of each strain were inoculated on PDA-coated glass slides. The Ca^2+^-free YEG medium (1 L YEG, 5 g yeast extract, 10 g glucose and 7 g agar) was used to test the response to high calcium ion stress.

### 4.2. Isolation and *Phylogenetic Analysis* of CgEnd3

The sequence of CgEnd3 (Protein ID: 1451078) was obtained from the genome database of *C. gloeosporioides* (http://genome.jgi.doe.gov/Gloci1/Gloci1.home.html) (accessed on 10 June 2019) using the BLASTP algorithm, based on the sequence of the *S. cerevisiae* End3p (NP_014315.1). The identification of CgEnd3 was based on the amino acid sequence homology to other fungi, including MoEnd3 in *M. oryzae* (MGG_06180), CoEnd3 in *C. orbiculare* (TDZ22908.1), CfEnd3 in *Colletotrichum fructicola* (XP_031885343.1), NcEnd3 in *Neurospora crassa* (XP_962381.2), FoEnd3 in *Fusarium oxysporum* (EXM25519.1), and End3p in *S. cerevisiae* (NP_014315.1) using ClustalX 2.1 (Figure S1A). The phylogenetic tree was constructed by MEGA 7.0 using full-length protein sequences and the neighbor-joining method with 1000 bootstrap replications (Figure S1B), as previously described [91]. The EF hand domain and calcium ion binding sites of CgEnd3 were predicted using the InterProScan tool (https://www.ebi.ac.uk/interpro) (accessed on 10 June 2019) [92].

### 4.3. Generation of CgEnd3 Deletion Mutants

The split-marker method [93] was used to construct targeted deletion cassettes containing the sulfonylurea (*Sur*) resistance gene to replace the native *CgEnd3* gene in *C. gloeosporioides* (Figure S2A). The *Sur* resistance-conferring gene, carried by PCB1523 [94], was kindly provided by Prof. Richard Wilson, University of Nebraska-Lincoln. Firstly, to generate the fusion fragments, approximately 1.5 kb upstream and downstream flanking sequences were amplified using the primer pairs CgEnd3-5Ffor (1F)/CgEnd3-5Frev(2R) and CgEnd3-3Ffor (2F)/CgEnd3-3Frev (3R), respectively. The full length of the *Sur* was amplified using primer pairs SUR-5′-M13F and SUR-3′-M13R, which included an approximately 20 bp sequence that overlaps (M13F or M13R) the PCR products of the two flanking sequences of *CgEnd3*. Second-round PCR was applied to generate the fusion constructs, and upstream and downstream flanking sequences were fused with two-thirds sulfonylurea resistance cassette, respectively.

To generate the deletion mutants of *CgEnd3*, PEG-mediated transformation was used [54]. The resulting transformants were first screened on solid TB3 medium with 150 μg/mL sulfonylurea, and the putative *CgEnd3*-deletion mutants were screened by PCR with the primers External-CgEnd3for (3F)/External-CgEnd3rev (4R) (Figure S2B) and primers Internal-CgEnd3for (4F)/Internal-CgEnd3rev (5R) (Figure S2C). For the complementation assay, the phleomycin resistance cassette (amplified from the pBC-phleo vector provided by FGSC) was used as a selective marker, and the entire coding sequence of *CgEnd3*, with approximately 1.5 kb upstream region, was transformed into the *CgEnd3* deletion mutant (Δ*CgEnd3-12*). The complementation mutants were screened using Internal-CgEnd3for (4F)/Internal-CgEnd3rev (5R) (Figure S2D). The complementation strain was described as Δ*CgEnd3*/END3 throughout this study. All primers used in this study are listed in Table 1.

### 4.4. Gene Expression Analysis

To analyze the expression of calcium-signaling genes and chitin synthase genes, the RNA of each strain was first extracted. Fresh hyphae from WT and Δ*CgEnd3* were cultured for 2 days using liquid CM under shaking environment at 25 °C. The total hyphae (300 mg) from each strain was dried with filter paper and powdered using liquid nitrogen. Total RNA was extracted using TRIzol reagent (Invitrogen), following the manufacturer’s instructions. To analyze the expression of core effectors during the early stage of infection, equal volumes (30 μL) of conidial suspensions (10^6^ conidia/mL) from WT and Δ*CgEnd3* were inoculated on poplar leaves. Inoculated leaves were fixed on filter paper and placed into a 94 mm Petri dish containing 8 mL of sterile water; sterile cotton wool wetted with sterile water was used to cover the petioles of the leaves. Petri dishes were incubated at 25 °C. The inoculation area, including fungal tissue and leaf tissue, were cut at 3 dpi, and 5 mg samples from WT and Δ*CgEnd3* area were collected and powdered using liquid nitrogen, respectively. Total RNA was extracted using TRIzol reagent (Invitrogen), following the manufacturer’s instructions. Then, 0.8% agarose gel was applied to test the RNA integrity. Additionally, cDNAs were synthesized using the FastKing cDNA synthesis kit (Tiangen, China). The *Cg18S* gene served as an internal reference in this study. The Bio-Rad CFX96 PCR system (Bio-Rad, Hercules, CA, USA) was used to analyze the expression of related genes in this study. Relative gene expression in this study was calculated using the 2^−ΔΔCT^ method [95]. Each experiment was performed three times.

### 4.5. Fungicide Response Assay

To test the response to fungicide, two broad-spectrum fungicides, difenoconazole and fludioxonil, were tested in this study. Difenoconazole and fludioxonil were dissolved in acetone at a concentration of 1 mg/mL. Hyphal blocks of each strain were inoculated on PDA mixed with difenoconazole (0.8 μg/mL) and fludioxonil (5 and 10 μg/mL), respectively. Plates were cultured at 25 °C. This experiment was performed three times.

### 4.6. Cellophane Membrane Penetration Assays

To test the penetration ability of each strain, hyphal blocks from each strain were inoculated on the sterile cellophane membrane overlaid on PDA plates. The cellophane membrane was cut into a size of 3 × 3 cm. Plates were cultured at 25 °C for 2 days (Pre). At 2 dpi, the entire membrane with colony was removed, and the resulting plates were cultured at 25 °C for 2 days (Post). This experiment was performed with three biological replicates and five technical replicates for each treatment.

### 4.7. Pathogenicity Assay

To determine the pathogenicity of WT and *CgEnd3* deletion mutant, the susceptible species *Populus × beijingensis* was used as a host throughout this study. Annual poplar branches were cultured in water and poplar leaves detached from two-week-old water-cultivated poplar branches. To remove microorganisms and dust on the surface of the leaves, poplar leaves were dipped in 75% ethyl alcohol for 10 s and thoroughly washed with sterile water. Equal volumes (30 μL) of conidial suspensions (2 × 10^5^ conidia/mL) from each strain were inoculated on poplar leaves. Inoculated leaves were fixed on filter paper and placed into a 94 mm Petri dish containing 8 mL of sterile water, and wettened sterile cotton wool was used to cover the petioles of the leaves. Petri dishes were incubated at 25 °C. Symptoms were pictured from 4–8 dpi. Lesion size was measured using quadrille paper. This experiment was performed with three biological replicates and five technical replicates for each treatment.

### 4.8. DAB and FM4-64 Staining

Conidia of the WT, Δ*CgEnd3*, and Δ*CgEnd3*/END3 were resuspended in sterilized deionized water (10^5^ conidia/mL). Conidial suspensions (30 µL) of each strain were inoculated on the hydrophobic surfaces of onion epidermal cells. Inoculated samples were fixed on filter paper and placed into a 94 mm Petri dish containing 8 mL of sterile water and incubated at 25 °C. DAB was used to detect the accumulation of ROS. In the presence of ROS, DAB is converted to dark brown polymers. At 9 hpi, the accumulation of ROS was stained with 2 mg/mL DAB (30 µL) for 12 h under darkness. The intensities of dark brown polymers were quantified using ImageJ software, and at least 10 appressoria were analyzed from each strain. This experiment was performed with three biological replicates and five technical replicates for each treatment.

FM4-64 (Thermo fisher, Waltham, MA, USA) was dissolved in sterile water at the final concentration of 5 µM. Hyphal block from WT, Δ*CgEnd3*, and Δ*CgEnd3*/END3 were inoculated on PDA-coated glass slides, and samples were incubated at 25 °C. Then, at 2 dpi, the radiate hyphae were stained with FM4-64. The samples were observed under a fluorescence microscope immediately. Excitation spectra: 535 ± 20 nm, emission spectra: 610 ± 30 nm. Images were pictured from 0 to 30 min. This experiment was performed with three biological replicates and five technical replicates for each treatment. To analyze the intensities of endocytosis, the fluorescence intensities in the cytoplasm at each of the time points were quantified using ImageJ software; at least 10 samples were analyzed from each strain. This experiment was performed with three biological replicates and five technical replicates for each treatment.

## Figures and Tables

**Table 1:** Primers used in this study.

Primer Name	Sequence	Use in This Study
CgEnd3-5Ffor (1F)	AGACTGGCCAAAAGTGTTCG	5F flanking sequence
CgEnd3-5Frev (2R)	CTCCCACAGGAATCTCCGTC	
CgEnd3-3Ffor (2F)	TACGCTTCGATACCCTTCGG	3F flanking sequence
CgEnd3-3Frev (3R)	CTTGGAGCGACAAGTTGGGA	
External-CgEnd3for (3F)	TTCCTAGCGACCCTGTTGTT	External sequence used for validation of mutant
External-CgEnd3rev (4R)	GCCCGGTGAGTAGAATGGTA	
Internal-CgEnd3for (4F)	AATAACAACCCCGCCTCTTC	Internal sequence used for validation of mutant
Internal-CgEnd3rev (5R)	AGCTGCTTCTTGAGCCTGAC	
CgEnd3-Compfor	TCGACTCGACAACATCAAGC	Complementary sequence
CgEnd3-Comprev	GCCCGGTGAGTAGAATGGTA	
SUR-5′-M13F	CGCCAGGGTTTTCCCAGTCACGACGTCGACGTGCCAACGCCACAG	Sur cassette contains the region of M13F and M13R
SUR-3′-M13R	AGCGGATAACAATTTCACACAGGAGTCGACGTGAGAGCATGCAAT	
SU-SPLIT	CCAAGCATGTGCAGTGCCTTC	The 2/3rd portion of the Sur cassette
UR-SPLIT	GGAGGCCGACGTCATAGGCATC	
CgPMC1-f	CATCATGATTGCTGGTCAGG	qRT-PCR of CgPMC1
CgPMC1-r	GACACCGAAAGGAATGGAAA	
CgNCS1-f	AGCGACAAGTCAGGAAGCAT	qRT-PCR of CgNCS1
CgNCS1-r	CCTCGACGATCTTGAGCATT	
CgCAM1-f	ACAACAACGGCTTCATCTCC	qRT-PCR of CgCAM1
CgCAM1-r	GCGAATCATCTCGTCAACCT	
CgCRZ1-f	AGGTCGGATCTGCATCAAAC	qRT-PCR of CgCRZ1
CgCRZ1-r	ATGTACTGGCCGCTGGTATC	
CgCAMK1-f	ATGCTGAAGAAGGGTCATGG	qRT-PCR of CgCAMK1
CgCAMK1-r	TGAAATCCTTGGCATCATCA	
CgCHS1-f	GGTGGTGGTCTGAAGCGTTA	qRT-PCR of CgCHS1
CgCHS1-r	AGCGCATCTTGTGGAACTCA	
CgCHS2-f	GACTACGCCCGCGAATATGA	qRT-PCR of CgCHS2
CgCHS2-r	GTTCGTAGATGCCGGAAGGA	
CgCHS3-f	CACCGGCTACAGCGAGTATG	qRT-PCR of CgCHS3
CgCHS3-r	CAAGTTTCCGCGGTACAGGA	
CgCHS4-f	CGACAAGGACCATCCGAACT	qRT-PCR of CgCHS4
CgCHS4-r	GCGAGGCATCGGAGTAGTTA	
CgCHS5-f	CTCAGGGCGGCATTGATACT	qRT-PCR of CgCHS5
CgCHS5-r	AGTAGAGCGTAGTTGGAGGC	
CgCHS6-f	ACCAATCCGGTGCTTATCGG	qRT-PCR of CgCHS6
CgCHS6-r	GGGGTCTCGAAGCCAAGATG	
CgCHS7-f	TGGTCAAGGGCCTTCAATGG	qRT-PCR of CgCHS7
CgCHS7-r	ATCACAACCTTTGGTGCGGA	
CgDN3-f	CCTACTCGCTGTTCCCTTCA	qRT-PCR of CgDN3
CgDN3-r	CGTGGTCTCCCGGATAGTAG	
CgEC6-f	TTGGCAGTATCACCGTGAAG	qRT-PCR of CgEC6
CgEC6-r	TCGATCTCATCCTGGTAGGC	
CgFl-f	GCTGTTGAGTCCGGTGGTAT	qRT-PCR of CgFl
CgFl-r	GGGTGGTGGTCATAGAGGTG	
CgNis1-f	ATCTACGGCATTGCCTTCC	qRT-PCR of CgNis1
CgNis1-r	AGAAGGAGCCGATGACTGTG	
Cg18S-f	GTGAGGCCCTCAAAGGTAGTGG	qRT-PCR of Cg18S
Cg18S-r	GGATCCCAGTGCGAGACGT	

## Data Availability

The data presented in this study are available in “CgEnd3 regulates endocytosis, appressorium formation and virulence in the poplar anthracnose fungus *Colletotrichum gloeosporioides*”.

## Supplementary Materials

The following are available online at https://www.mdpi.com/1422-0067/22/8/4029/s1.
