# Supplementary figures and images for: CgEnd3 Regulates Endocytosis, Appressorium Formation, and Virulence in the Poplar Anthracnose Fungus Colletotrichum gloeosporioides
Source: Int J Mol Sci. 2021 Apr 14;22(8):4029. doi: 10.3390/ijms22084029 (PMC8103510; doi:10.3390/ijms22084029)

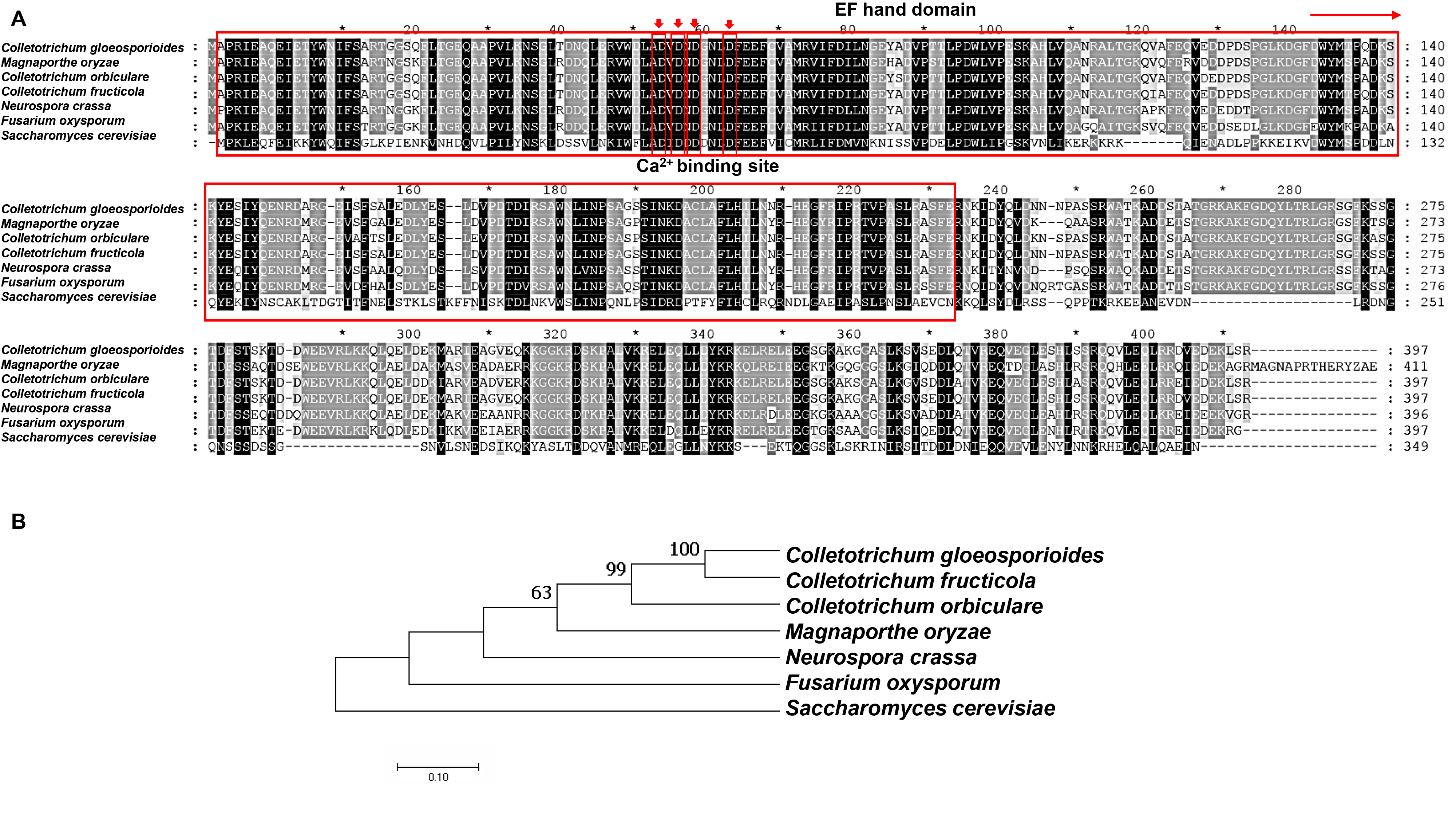

Supplement: Supplementary file 1 [file ijms-22-04029-s001.zip › Supplementary files/Figure S1.tif]

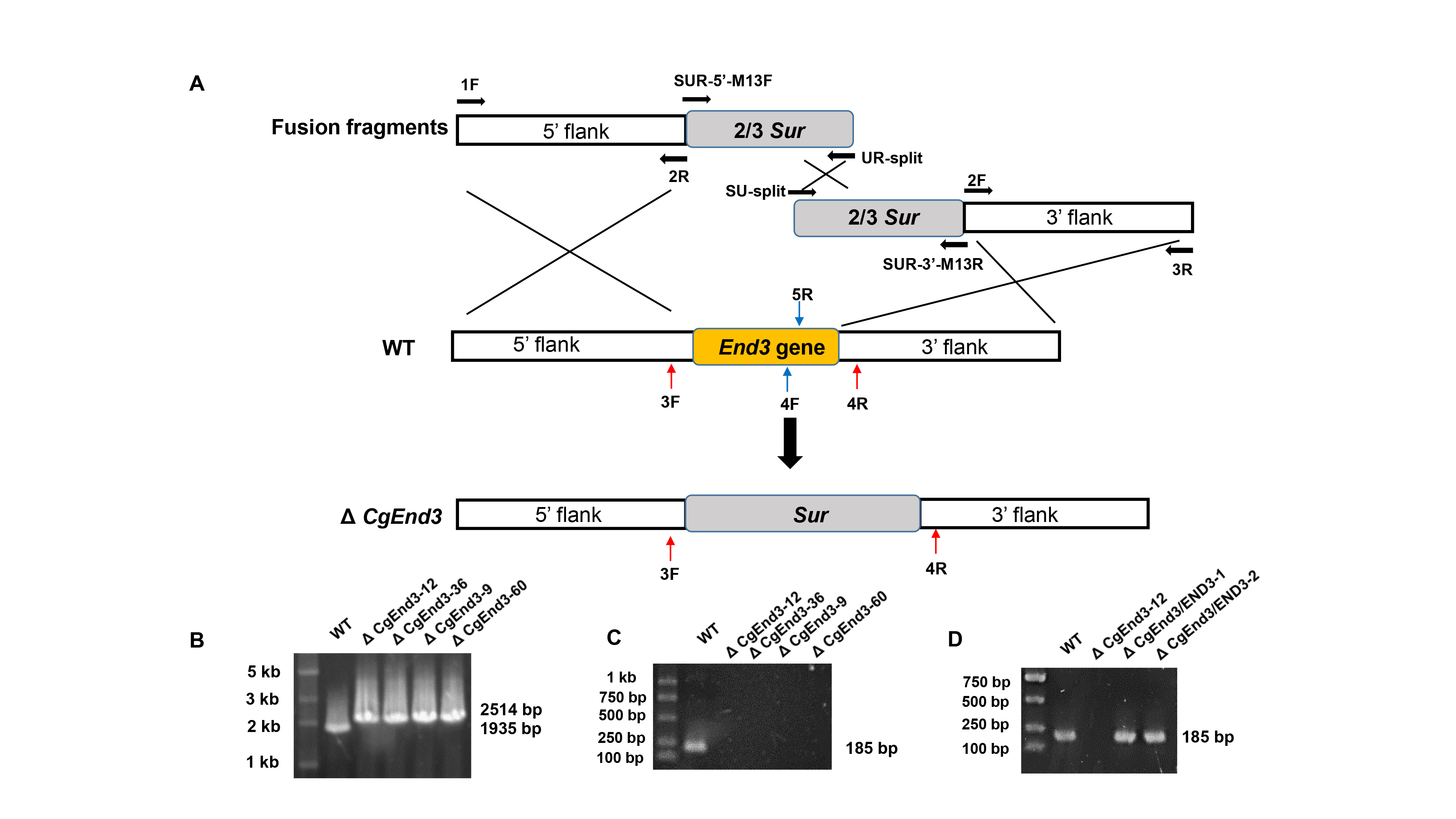

Supplement: Supplementary file 1 [file ijms-22-04029-s001.zip › Supplementary files/Figure S2.tif]
